# New Biodegradable Materials for Re-Thought Packaging from Pre-Consumer Wastes by Controlling the Storage Time as Method to Increase the Mechanical Recycling Efficiency

**DOI:** 10.3390/ma16041503

**Published:** 2023-02-10

**Authors:** Doina Dimonie, Mihail Dragne, Bogdan Trica, Cristian-Andi Nicolae, Monica Raduly, Sanda Doncea, Magda Ladaniuc, Alina Mustatea, Florentina Miu, Laurentiu Soare, Tudor Georgescu

**Affiliations:** 1 Chemical Engineering and Biotechnologies Faculty’ Doctoral School, University Politehnica of Bucharest, 313 Splaiul Independentei, 060042 Bucharest, Romania; 2 National Institute for Research and Development in Chemistry and Petrochemistry, 202 Splaiul Independentei Street, 060021 Bucharest, Romania; 3 PROMATERIS SA, Bucharest-Targoviste Street, No.1, 070000 Buftea, Romania

**Keywords:** starch, PCL, PBAT, enzymatic degradation, storage, compositional de-mixing

## Abstract

The influence of storage conditions on the mechanical recycling of pre-consumer waste (PRE-CW) from the manufacture of multilayer packaging films starting from starch compounds using a renewable-based polymer with PCL and PBAT, which are biodegradable conventional-based polyesters, was studied. It was found that, unlike materials based on conventional-origin polymers that accumulate in the environment for hundreds of years, the studied compounds degraded, even in the solid state, duringstorage in unventilated spaces and during the rainy hot summers with alternatingheat and rain. The degradation of the mechanically recycled compounds obtained from PRE-CW stored in such conditions was highlighted by the comparative analysis with the primary compounds, which proved the following: specific FTIR spectra changes; 2–3-times higher melt fluidity than for primary compounds; melting in successive processes over the entire positive temperatures range, up to 115 °C, such as in cases of compositional de-mixing of incompatible blends, faced to a single melting endotherm with a maximum at around 120 °C for the primar thermal degradation with the movement of the main destruction stages towards higher temperatures; a high quantity residue at 750 °C in air; dispersed mechanical resistance properties y compounds; crystallization at temperatures 10 °C–15 °C higher. The elimination of storage before the mechanical recycling of the pre-consumer waste from this type of polymeric compound fabrication is a way to increase the mechanical recycling efficiency while obtaining new materials with functional properties required by the applications.

## 1. Introduction

The connection to a production and consumption model particular to the circular economy is also based on the intelligent, innovative and sustainable manufacturing of plastic materials that respects the needs of reduction at the source, as well as reuse, repairing and recycling. The mechanical recycling of the pre-consumer waste (PRE-CW) resulting from the manufacture of polymer products,in addition to the post-consumer waste (POST-CW),which is obtained after the life end of the plastic items, represents a good solution to developing a sustainable re-thought packaging fabrication for integration into the circular economy [1,2,3,4]. It is no longer necessary to specify that the circular economy involves the manufacturing of materials based on conventional polymers as well as those of renewable origin [5,6,7]. The lack of structural damage at the life end of secondary polymers coming from PRE-CW or POST-CW has generated the belief that they can be considered true deposits of raw materials, which apparently show a greater or lesser content of non-polymeric impurities, depending on the field in which they were used. However, there are also situations in which, after the life end, the polymers have modified chemical structures due to chemical aging during use. It is believed that the PRE-CW from the synthetic polymers manufacture can be mechanically recycled effectively because if they have been properly melt-processed into finished products, then they are not chemically degraded, and if they have been selectively collected, then they are similar to primary polymers [8,9].

The solid-state chemical degradation of the conventional polymer-based materials is mainly related to biodegradation and only works in very few cases, the proof being their environmental accumulation from the perspective of their degradation over hundreds of years [10]. The diminishment of the functional properties of the multi-component compounds based on conventional-origin polymers can also be due to physical aging generated by macromolecule relaxation until reaching the least energetically charged conformation [11,12,13,14,15,16]. In practical, this aspect is reflected by the warranty period required of any manufacturer as the period in which the thermodynamic equilibrium is stable and therefore the functional properties are preserved [17]. There are known studies which, to ensure the useful life by increasing the thermodynamic stability, deal with melt structuring techniques, which make miscibility improvement possible at the molecular level via increasing the dispersion degree of the minority components into the majority matrix of starch, e.g., [18,19,20].

As it is known, certain renewable polymers and a few conventional-origin polyesters are biodegradable [21].In this context, knowing that, even in the solid state, chemical destruction as well as physical aging can occur, the question of whether the PRE-CW from the manufacture of starch and conventionally biodegradable polyester compounds can be stored, and if so, for how long and under what conditions, before mechanical recycling is legitimate. This question deserves an answer, especially from a practical point of view, since it is possible that during storage, depending on the environmental characteristics in which they are kept, due to multiple possible transformations of a physical and chemical nature, compositional de-mixing may occur with a drastic change in functional properties, possibly with the entire loss of the polymer material characteristics, causing their mechanical recycling as a source of raw materials to be impractical. Otherwise, this question is legitimate for all materials containing polymers of renewable origin [22]. Existing information related to the storage stability of such materials was not found or was generally non-specific [23].

The aim of this article was to study the influence of storage conditions on the mechanical recycling efficiency of some pre-consumer waste from the manufacture of multilayer packaging films and from bags and sacks obtained from these films, starting from compounds based on starch of renewable origin and two biodegradable polyesters of conventional origin, PCL and PBAT, and to identify the PRE-CW waste storage conditions that make it possible to efficiently increase mechanical recycling and obtain new biodegradable materials with secondary polymer contents and the required functional properties for the desired application.

## 2. Materials and Methods

### 2.1. Methods

**Figure 1:**
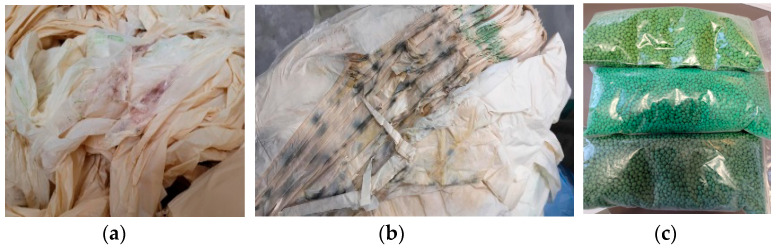
Mechanical recycling of PRE-CW from film fabrication starting from starch, PCL and PBAT compounds in the same films for packaging manufacturing (**a**,**b**) PRE-CW stored in uncontrolled conditions; (**c**) pellets mechanically recycled from PRE-CW.

The PRE-CW (Figure 1) from the manufacture of multilayer films (three equal layers from the same compound) and from the bags and sacks of these films (Figure 1a),some of which developed microorganisms (Figure 1b) due to being stored during hot summers with alternating heat and rain episodes in spaces without ventilation or temperature control, were separated by color and then recycled mechanically (Figure 1c) via specific working phases of sorting, transport, shredding, extrusion–granulation, drying, packaging, and storage. The PRE-CW was stored for 4 months (120 days), from June until the end of September, in an unventilated room (indoor environment), at T 30 °C–75 °C and 15–70% relative humidity. The mechanically recycled pellets were compounded with primary pellets on a classic installation that contained a single screw extruder with 17 L/D under usual working conditions (temperature profile: barrel of 150 °C, 155 °C, 160 °C, 180 °C, 170 °C, 170 °C and 170 °C, and die of 165 °C–170 °C) at an economically blended ratio, and later, the newly obtained pellets were used in the same packaging film manufacturing.

In order to identify the influence of PRE-CW storage in non-ventilated spaces, under the action of temperature and humidity generated by evaporation during hot and rainy summers, in the performed degradation study, the properties in the molten and solid states of the mechanically recycled pellets were comparatively analyzed with those of the primary pellets used in the film manufacturing from which the mentioned wastes had resulted.

The degradation in the molten state was assessed by analyzing the dependence of the fluidity on the flow conditions and in the solid state by following the FTIR spectral changes, analyzing the thermal behavior estimated by DSC and TGA measurements and thetensile resistance of films with recycled compound content.

The results of the measurements regarding the fluidity of the melts were processed in such a way that an easy comparison was possible between the primary pellets and the mechanically recycled ones.

Thus, melt fluidity variation increments, ΔMFI, with the flowing conditions in two variants were calculated (Equations (1) and (2)). ΔMFI_T_ describes the variation of fluidity with temperature at constant weight and is equal to the difference between the melt fluidity at two successive temperatures and the same load (Equation(1)), and ΔMFI_kg_ describes the variation of melt fluidity with load at constant temperature and is equal to the difference between the melt fluidity at two successive loads at the same temperature (Equation (2)) [24,25,26].

(1) $${\Delta \mathrm{MFI}}_{T}{=\mathrm{MFI}}_{T,i}{-\Delta \mathrm{MFI}}_{T,i-1}$$

(2) $${\Delta \mathrm{MFI}}_{\mathrm{kg}}{=\mathrm{MFI}}_{\mathrm{kg},i}{-\Delta \mathrm{MFI}}_{\mathrm{kg},i-1}$$

### 2.2. Materials

The mechanically recycled PM2, PM3, and PM4 (Table 1) and primary F2, F3, and F4 (Table 2) compounds were used. The PM2, PM3, and PM4 represent PET-CW from the production of films manufactured from blends coded as F1, F2, and F3, after mechanical recycling. The F2, F3 and F4 were blends based on starch (S)—a copolymer poly(butylene adipate-co-terephthalate) comprising adipic acid, 1, 4-butanediol and dimethyl terephthalate (T_m_ = 267 °C–276 °C)—as the main component in all studied variants and polycaprolactone (PCL) used in a proportion of (30–60)% faced to 100% starch (T_m_ = 106 °C, T_degr_ = 140 °C–230 °C) and layered silicate (T_m_ = 60 °C).

### 2.3. Characterization

#### 2.3.1. Rheological Properties in the Melted State

For the rheological properties in the melted state, ISO 1133:2012 was used to compare measurements performed for mechanical and primary pellets, under the same flow conditions. Indexer Dynisco Polymer Test—LMI 4000: temperature range 50 °C–450 °C, temperature measurement accuracy ≤0.2 °C, temperature fluctuation ±0.1 s, time precision ±0.1 s, nozzle l/d = 2.09, temperatures between T = 130 °C–170 °C, and indexer loads of 2.16/3.8/5/10 kg.

#### 2.3.2. DSC

DSC Q2000 (TA Instruments, New Castle, DE, USA), RCS-90, pan T zero aluminum 100 µL; purge gas: helium (99.999%) 25 mL/min; method: equilibrate at −60 °C, isothermal for 3 min, ramp 10 °C/min to 200 °C, isothermal for 3 min, ramp 10 °C/min to 200 °C, isothermal for 3 min, ramp 10 °C/min to −60 °C, isothermal for 3 min, then ramp 10 °C/min to 250 °C. To eliminate the thermal history of each sample [27], the DSC was performed in cycles with two heating ramps and a cooling one. The melting and the glass transition were described using the second heating thermogram and the crystallization of those registered during cooling between the two heating runs.

#### 2.3.3. TGA

Q5000IR (TA Instruments), pan Platinum 100µL;purge gas 1: nitrogen (99.999%) 50 mL/min;purge gas 2: synthetic air (99.999%) 50 mL/min; method: ramp 10 °C/min to 750 °C, select gas 2, then isothermal for 5 min; T_max_ ( °C) = T(dα/dt) max.

#### 2.3.4. FTIR

Jasco FTIR 6300 spectrometer (Jasco Corporation, Tokyo, Japan) equipped with an ATR Specac Golden Gate (KRS5 lens), in the range 400–4000 cm^−1^ (32 scans at 4 cm^−1^ resolution). The normalization of all spectra was achieved by relating the intensity of each absorption to that of the highest of those found at 1714 cm^−1^, attributed to the C=O group vibration. The normalized spectra are presented in the Supplementary Materials.

#### 2.3.5. Mechanical Characterization

Mechanical characterization: tensile strength at break (*σ_br_*); elongation at break (ε); ISO 527-3:2018; 23 °C; RH = 50%; speed = 500 mm/min; pressure = 4 bar; Instron E2-F1-G1, with load cell of 100 N; specimen type 5 obtained from film by die-cutting and 5 repetitions for each condition. The standard deviation was calculated for each situation and represented graphically.

## 3. Results

### 3.1. Melt Rheological Properties

**Figure 2:**
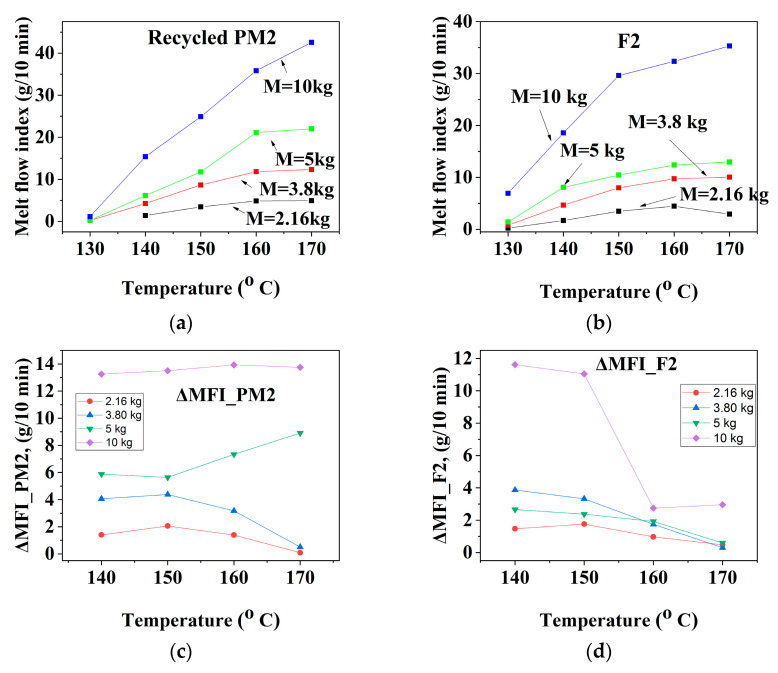

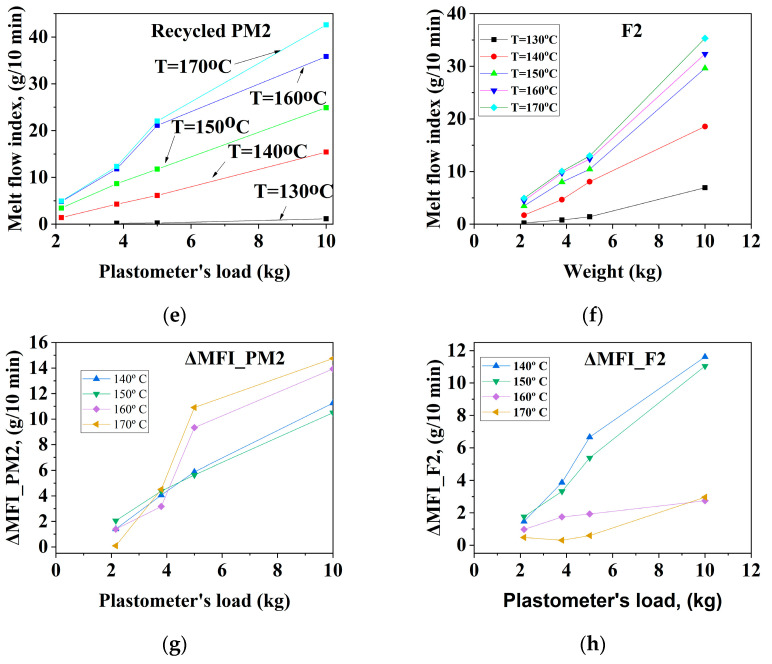
The melt rheological properties of the recycled **PM2** compound (**a**–**d**) with those of the primary **F2** (**e**–**h**) and their dependence on the flowing conditions (for PM2: (**a**) MFI = f(T); (**b**) ΔMFI = f(T); (**c**) MFI = f(G); (**d**) ΔMFI = f(G)); (for **F2**: (**e**) MFI = f(T); (**f**) ΔMFI = f(T); (**g**) MFI = f(G); (**h**) ΔMFI = f(G)).

**Figure 3:**
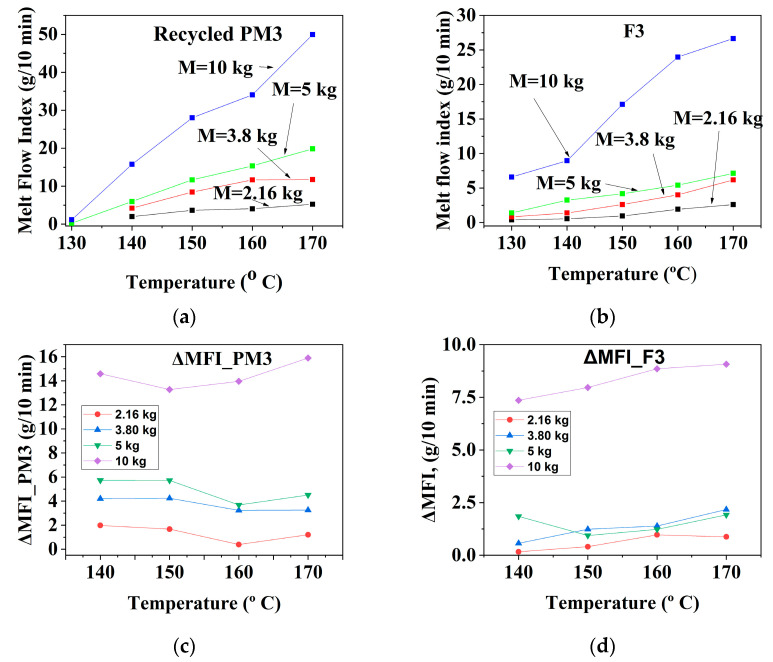

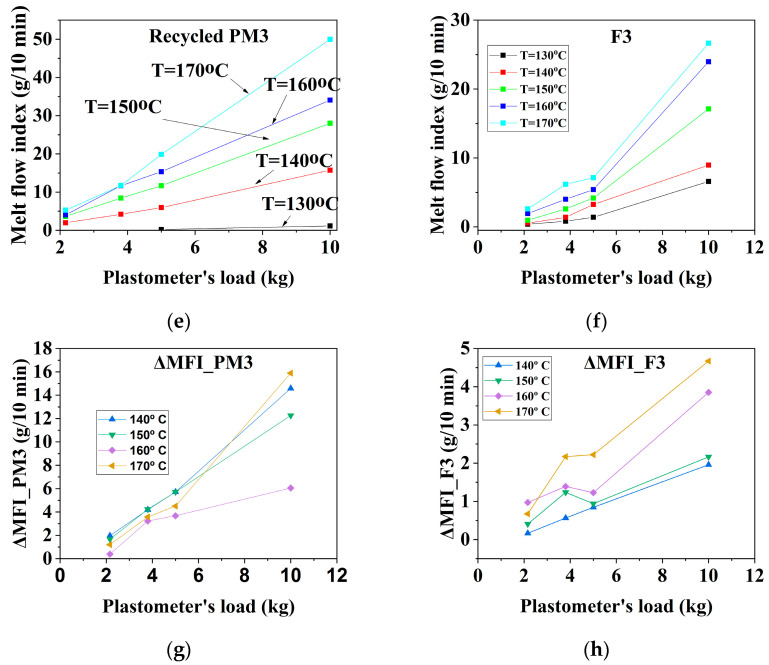
The melt rheological properties of the recycled **PM3** compound (**a**–**d**) with those of the primary **F3** (**e**–**h**) and their dependence on the flowing conditions (for PM3: (**a**) MFI = f(T); (**b**) ΔMFI = f(T); (**c**) MFI = f(G); (**d**) ΔMFI = f(G)); (For F3: (**e**) MFI = f(T); (**f**) ΔMFI = f(T); (**g**) MFI = f(G); (**h**) ΔMFI = f(G)).

**Figure 4:**
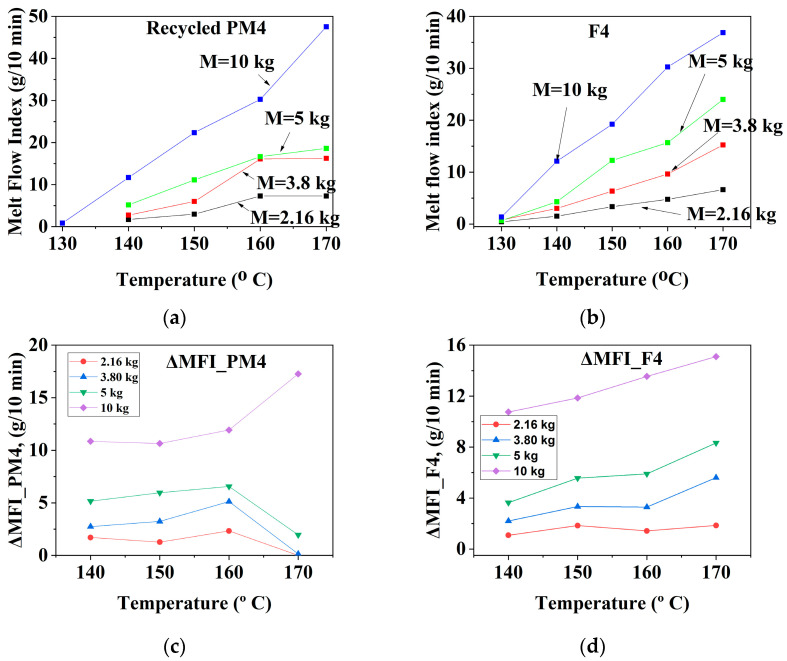

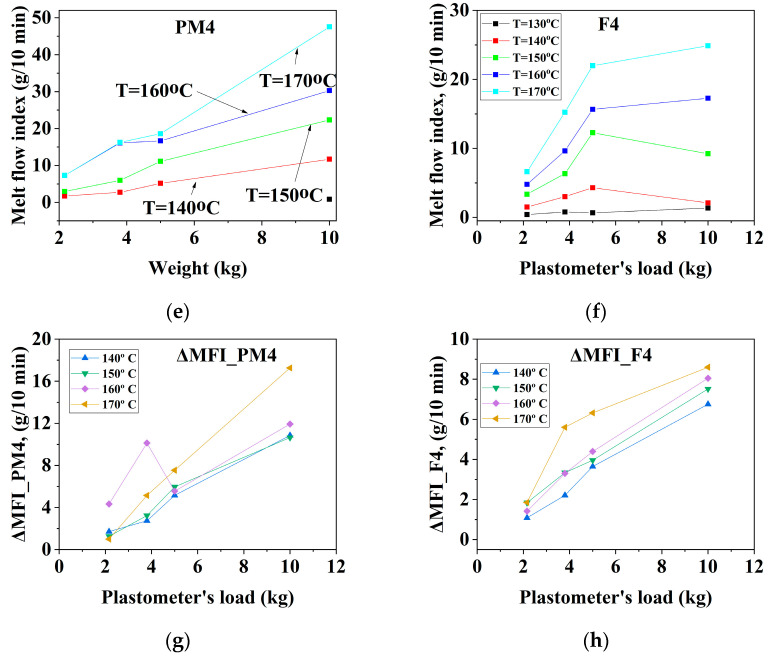
The melt rheological properties of the recycled **PM4** compound (**a**–**d**) with those of the primary **F4** (**e**–**h**) and their dependence on the flow conditions (for PM4: (**a**) MFI = f(T); (**b**) ΔMFI = f(T); (**c**) MFI = f(G); (**d**) ΔMFI = f(G)); (for F4: (**e**) MFI = f(T); (**f**) ΔMFI = f(T); (**g**) MFI = f(G); (**h**) ΔMFI = f(G)).

The results are presented graphically in Figure 2, Figure 3 and Figure 4 and summarized in Table 3, and their analysis is described below.

#### 3.1.1. Mechanically Recycled Compounds from the PET-CW Resulted from Manufacture of Films Flexographically Printed with Black (PM2) and Primary Compound (F2) Used in this Production

At a temperature between 160 °C and 170 °C and up to a max of 5 kg of the plastometer loads, the ΔMFI_T_ fluidity increment characterizing the melt flow of the **PM2** pallet (Figure 2a) ranged from 0.5–4 g/10 min, and between 140 °C and 150 °C it ranged from11 g/10 min–12 g/10 min (Figure 2b, Table 3). At temperatures of T = 160 °C–170 °C and loads between 2.16 kg and 10 kg, the variation of the ΔMFI_kg_ (Figure 2c, Table 3) ranged from 0.5–2 g/10 min (Figure 2d, plates 3), and at T = 150 °C–160 °C and 3.8–10 kg, this increment varied between 5 g/10 min and 12 g/10 min (Figure 2d).These results prove that the PM2 compounds must be processed at high temperatures and low loads.

At loads from 2.16 kg to 5 kg, the ΔMFI_T_ fluidity increment of primary compound **F2** (Figure 2e) had values between 1 g/10 min and 8 g/10 min, and at 10 kg load this increment became14 g/10 min (Figure 3f, Table 4). For this compound, the fluidity increment (Figure 2g, Table 4) at 10 kg was between 0.5 g/10 min and 2 g/10 min at 160 °C–170 °C temperature, and2.16 kg–5 kg ranged from2 g/10 min–12 g/10 min for temperatures between 140 °C–150 °C (Figure 2h, Table 4). It follows that the F2 compounds must be melt-processed at high temperatures and low loads.

#### 3.1.2. Mechanically Recycled Compound from PET-CW Formed during the Manufacture of Unprinted Films (PM3) and Primary Compound (F3) Used in this Production

Between 140 °C and 170 °C and loads between2.16 kg and 5 kg, the ΔMFI_T_ fluidity increment for the mechanically recycled **PM3** compound was between 2 g/10 min–6 g/10 min, and between 13 and 15 g/10 min at a load of 10 kg (Figure 3c, Table 3). For this compound, the ΔMFI_kg_ fluidity increment increased continuously from 2 to 16 g/10 min throughout the entire range of loads from 2.16 kg to 10 kg, with small differences dependent on the temperature value (Figure 3g, Table 4).

The ΔMFI_T_ fluidity increase for compound **F3** over the entire studied temperature range of 140 °C–170 °C and between loads of 2.16 kg and5 kg ranged from 0.2 g/10 min–2 g/10 min, and at 10 kg ranged from7.5 g/10 min–9.5 g/10 min (Figure 3d, Table 3).The ΔMFI_kg_ fluidity increment characterizing the flow of the primary F3 compound varied over the entire range of temperatures (140 °C–170 °C) and loads (2.16 kg–10 kg) from 0.5 g/10 min to 4 g/10 min, with slightly higher values at temperatures of 160 °C–170 °C (Figure 3h, Table 3).The compound must be melt-processed at low and medium temperatures and loads.

#### 3.1.3. Mechanically Recycled Compounds from Pre-Consumer Waste from the Mass Manufacture of Green-Colored Film (PM4) and Primary Compound (F4) Used in this Production

**Figure 5:**
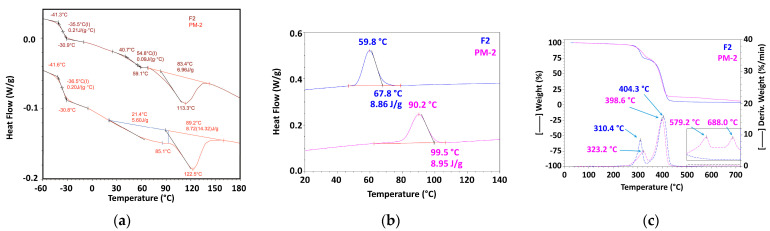
The thermal behavior of mechanically recycled (PM2) and primary compounds (F2) ((**a**) melting from second heating run; (**b**) crystallization; (**c**) TGA).

The ΔMFI_T_ fluidity increment for the **PM4** compound (Figure 5a,c) ranged from 1 g/10 min–6 g/10 min at loads between 2.16 kg and 5 kg and from11.5 g/10 min–18 g/10 min for the 10 kg load (Figure 4c, Table 3). The temperature-dependent fluidity increase for the F4 pellets had values between 0.5 g/10 min–5.5 g/10 min for loads between 2.16 kg–5 kg and 11 g/10 min–15 g/10 min for the10 kg load (Figure 4g). That is why this mechanically recycled compound must be melt-processed at low or medium temperatures and mechanical stress. The variation of the weight-dependent fluidity increment for **F4** pellets is between 0.9 and 6 g/10 min for loads in the range of 2.16 kg–5 kg and 5–8.5 g/10 min at 10 kg load (Figure 4h). The ΔMFI_kg_ fluidity increment for F4 compound is between 0.9 and 6 g/10 min for loads between 2.16 kg and 5 kg and of 5 g/10 min–8.5 g/10 min for 10 kg load (Figure 4h). That is why this compound must be melt-processed at high temperatures and low and medium loads.

The values of all the above-described fluidity increments for all the analyzed compounds, both primary and mechanically recycled, are centralized in Table 3.

The data presented in Table 3 shows that the fluidity increments of the mechanically recycled compounds from PRE-CW were2–5-times higher than those particular to the primary compounds used in the film manufacturing from which these wastes resulted. The greatest increase in the fluidity increments was recorded both for recycled and primary compounds at high loads (10 kg), even if the temperature values were low, which means that neither of them should be melt-processed at high shear. The melt-processing conditions must be individually identified for each type of compound. The flexographic printing and bulk coloring did not induce spectacular differences between the fluidity indices of the mechanically recycled compounds and the primary ones, possibly due to the small amounts of dyes used. In both cases, the fluidity indices were almost the same as for an un-dyed compound. The differences between the higher values of the fluidity increments for the mechanically recycled compounds compared to the primary ones was probably due tothe PRE-CW degradation during storage in uncontrolled conditions through a macromolecular chain scission mechanism. It is possible that during mechanical recycling the degradations were accentuated.

### 3.2. Thermal Behavior

#### 3.2.1. Mechanically Recycled Compounds from Pre-Consumer Waste Formed fromthe Manufacture of Films Flexographically Printed with Black (PM2) and Primary Pellets Used in this Production (F2) (Figure 5, Table 4, Table 5, Table 6, Table 7, Table 8, Table 9 and Table 10)

Comparing the thermal behavior of the recycled PM2 and primary F2 compounds, the critical differences were apparent, both regarding the main transitions (DSC) and the variation of weight loss with temperature (TGA). In the range of positive temperatures, the F2 compound presented a glass transition between 42.4 °C–60 °C (Figure 5a, Table 4) and a wide melting that starts at 83.4 °C. It had a maximum at 113.3 °C and ended at 145 °C with a thermal effect of 6.96 J/g (Figure 5a, Table 5). In contrast with F2, the mechanically recycled PM2 compounds did not have a glass transition and behaved as a de-mixed blend with two almost consecutive melts over the entire positive temperature range, up to 122 °C. The first started at 21 °C, with a maximum at 85.1 °C and a caloric effect of 5.6 J/g, followed by the subsequent melt starting at 89.2 °C, with a maximum at 122.5 °C and a thermal effect of 8.72 L/g (Figure 5a, Table 6). Comparing the characteristics of the glass transition of the primary compounds F2 (Figure 5a, Table 4) and those of the mechanically recycled PM2 (Figure 5a, Table 6), which was produced in the negative temperature range, it can be assumed that they were just about similar. The process started at −41 °C and ended at approx. −31 °C; T_g_ had a value of −36.5 °C with a ΔC_p_ of 0.21 g∙°C for F2 and 35.5 °C with a ΔC_p_ of 0.21 g∙°C for PM2. Differences between the PM2 and F2 compounds’ thermal behavior relating to the crystallization of these two blends were also recorded (Figure 5b, Table 7 and Table 8). The crystallization of the primary compound F2 took place through a relatively narrow and deep peak that started at 67.8 °C, with a maximum at 59.8 °C and a caloric effect of 8.86 J/g (Figure 5b, Table 8),and the mechanically recycled compound PM2 had a slightly wider crystallization exothermic peak and began to crystallize at 99.5 °C with a maximum at 90.2 °C and a caloric effect of 8.95 J/g (Figure 5b, Table 7).The TGA behavior also differentiated the two compounds F2 and PM2 (Figure 5c, Table 9 and Table 10). The primary F2 compound (Figure 5c, plate 9) lost 1.5% of its mass at 109.5 °C, then massively degraded in two stages, one between 175 °C and 340 °C with a maximum at 310.4 °C at which it lost 25.55%, and the next in the range of 340 °C–465 °C with a maximum at 404.3 °C at which it lost 68.64% of the total weight. This compound lost a little more at higher temperatures, namely 0.81% between 465 °C and 530 °C and 0.44% from 530 °C to720 °C. The residue recorded at 720 °C was4.05% in nitrogen and 0.24% in air. In comparison to the primary compound, the mechanically recycled compound PM2 had its own TGA behavior (Figure 5, Table 10). This compound lost 1.77% at 175 °C, a slightly higher temperature than for F2, then it massively degraded in two steps, similar to F2, but with slightly different percentages and temperatures at which those degradation steps took place. The mechanically recycled compound PM2 lost 23.38% of its weight in the range of 175 °C–345 °C with a maximum at 323.2 °C, and 62.52% between 345 °C and 485 °C with a maximum at 398.6 °C. Two other weight losses followed, one of 3.72% between 485 °C and 630 °C with a maximum at 579.2 °C and another of 3.10% from 630 °C to 750 °C with a maximum at 688 °C. The residue at 750 °C corresponded to the mechanically recycled compound PM2, and was 5.51% in nitrogen and 5.49% in air.

#### 3.2.2. Mechanically Recycled Compounds from Pre-Consumer Waste Formed during the Manufacture of Non-Printed Films (PM3) and Primary Compound (F3) Used in this Production (Figure 6, Table 4, Table 5, Table 6, Table 7, Table 8, Table 9 and Table 10) 

**Figure 6:**
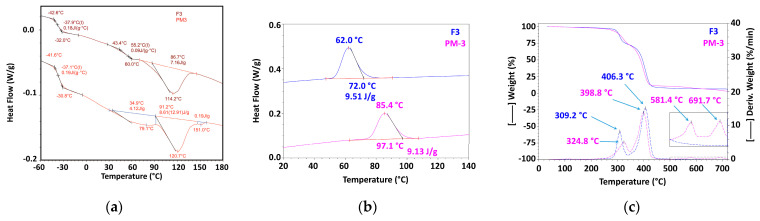
The thermal behavior of mechanically recycled (PM3) and primary compounds (F3) ((**a**) melting from second heating; (**b**) crystallization; (**c**) TGA).

**Figure 7:**
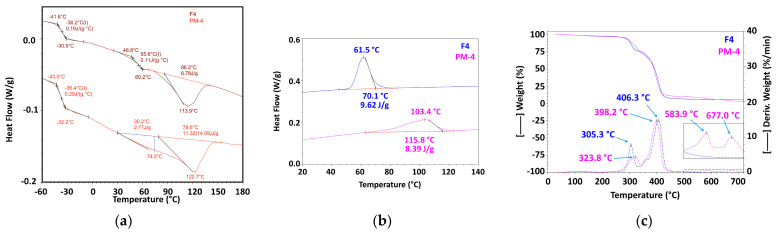
The thermal behavior of mechanically recycled (PM4) and primary compounds (F4) ((**a**) melting from second heating run; (**b**) crystallization; (**c**) TGA).

The primary compound F3 had a glass transition at 55.2 °C, which started at 43.4 °C and ended at 60 °C (Figure 6a, Table 4), but the mechanically recycled compound PM3 didnot show such a transition. The primary compound F3 had a single melting with a maximum at 114.2 °C, which started at 86.7 °C and consumed 7.16 J/g (Figure 7a, Table 5). By comparison, the similar mechanically recycled compound (PM3) had, over the entire analyzed temperature range, two melts of different intensity, which seemed to occur successively. The first melt had its maximum at 79.1 °C, started at 34.9 °C and consumed 4.12 J/g. The second melt had a maximum at 120.7 °C, started at 91.2 °C and needed twice much caloric energy, specifically 8.61 J/g (Figure 6a, Table 6). Comparing the characteristics of the glass transition of the primary compounds F3 (Figure 6a, Table 4) and those of the mechanically recycled PM3 (Figure 6a, Table 6), which was produced in the negative temperature range, it can be assumed that they were just about similar. The process started at −42 °C, ended at approx. −31 °C, with T_g_ having a value of −37.5 °C and ΔC_p_being 0.18 g∙°C for F3, and 37.1 °C with a ΔC_p_ of 0.19 g∙°C for PM3. The crystallization of the two compounds, the primary and the mechanically recycled one, proceeded differently (Table 7 and Table 8). The primary compound started to crystallize at 72 °C, had a maximum at 62 °C and released 9.51 J/g (Figure 6b, Table 8), while the mechanically recycled PM3 started to crystallize at 97.1 °C, had a maximum at 85.4 °C and released 9.13 J/g (Figure 6b, Table 7), which means a 23 °C higher crystallization maximum for the recycled material. The comparative analysis of the TGA behavior of the two compounds F3 and PM3 also showed differences between them (Figure 6c, Table 9 and Table 10). The compound F3 lost 1.5% up to 175 °C with a maximum at 109.9 °C. The important weight losses of this blend occurred in the following two steps: 25.37% in the first step, between 175 °C and 340 °C with a maximum at 309.12 °C, and 65.03% in the next step, from 340 °C to 465 °C with a maximum at 406.3 °C. Between 530 °C and 720 °C, F3 lost 1.26% and 0.75% between 530 °C and 720 °C. The residue at 720 °C was 2.35% in nitrogen and 2.19% in air.

**Table 4:** Glass transition of studied primary compounds.

2nd Heating	Glass Transition 1				Glass Transition2			
	Onset	T_g_	End	ΔC_p_	Onset	T_g_	End	ΔC_p_
	°C	°C	°C	J/(g·°C)	°C	°C	°C	J/(g·°C)
F2	−41.3	−35.5	−30.9	0.21	40.7	54.8	59.1	0.09
F3	−42.6	−37.9	−32.0	0.18	43.4	55.2	60.0	0.09
F4	−41.6	−36.2	−30.9	0.19	46.8	55.6	60.2	0.11

**Table 5:** Melting of studied primary compounds.

2nd Heating	Enthalpy 2		
	Onset2	T_m2_	ΔH_2_
	°C	°C	J/g
F2	83.4	113.3	6.96
F3	86.7	114.2	7.16
F4	86.2	113.9	6.78

**Table 6:** Melting of mechanically recycled compounds.

2nd Heating	Glass Transition				Enthalpy 1			Enthalpy 2			ΔH_1_ + ΔH_2_
	Onset	T_g_	End	ΔC_p_	Onset1	T_m1_	ΔH_1_	Onset2	T_m2_	ΔH_2_	
	°C	°C	°C	J/(g·°C)	°C	°C	J/g	°C	°C	J/g	J/g
PM2	−41.6	−36.5	−30.8	0.20	21.4	85.1	5.60	89.2	122.5	8.72	14.32
PM3	−41.6	−37.1	−30.8	0.19	34.9	79.1	4.12	91.2	120.7	8.61	12.72
PM4	−43.0	−36.4	−32.2	0.20	30.2	74.5	2.77	78.8	122.7	11.32	14.09

**Table 7:** Cooling of mechanically recycled compounds.

Cooling	Crystallization		
	Onset	Tc	ΔHc
	°C	°C	J/g
PM2	99.5	90.2	8.95
PM3	97.1	85.4	9.13
PM4	115.8	103.4	8.39

**Table 8:** Cooling of studied primary compounds.

Cooling	Crystallization		
	Onset	Tc	ΔHc
	°C	°C	J/g
F2	67.8	59.8	8.86
F3	72.0	62.0	9.51
F4	70.1	61.5	9.62

**Table 9:** Thermal degradation of primary compounds (TGA measurements).

Code/Prop	RT-175 °C		175–340 °C		340–465 °C		465–530 °C	530–720 °C	Residue at 720 °C	
	Wt. Loss	T_max_	Wt. Loss	T_max_	Wt. Loss	T_max_	Wt. Loss	Wt. Loss	N_2_	Air
	%	°C	%	°C	%	°C	%	%	%	%
F2	1.50	108.9	24.55	310.4	68.64	404.3	0.81	0.44	4.05	0.24
F3	1.35	128.1	25.37	309.2	65.02	406.3	1.26	0.77	6.23	0.41
F4	2.31	118.4	22.90	305.3	67.43	406.3	1.11	0.67	5.58	0.37

**Table 10:** Thermal degradation of mechanically recycled compounds.

Code/Prop.	RT—175 °C	175 °C–345 °C		345 °C–485 °C		485 °C–630 °C		630 °C–750 °C		Residue at 750 °C	
	Wt. Loss	Wt. Loss	T_max_	Wt. Loss	T_max_	Wt. Loss	T_max_	Wt. Loss	T_max_	N_2_	Air
	%	%	°C	%	°C	%	°C	%	°C	%	%
PM2	1.77	23.38	323.2	62.52	398.6	3.72	579.2	3.10	688.0	5.51	5.49
PM3	1.80	25.54	324.8	62.14	398.8	4.21	581.4	3.96	691.7	2.35	2.19
PM4	1.89	22.82	323.8	64.96	398.2	4.70	583.9	3.53	677	2.10	1.98

#### 3.2.3. Mechanically Recycled Compound from Pre-Consumer Waste Generated fromthe Mass Manufacture of Green-Colored Films (PM4) and Primary Compound (F4) Used in this Production (Figure 7, Table 4, Table 5, Table 6, Table 7, Table 8, Table 9 and Table 10) 

**Figure 8:**
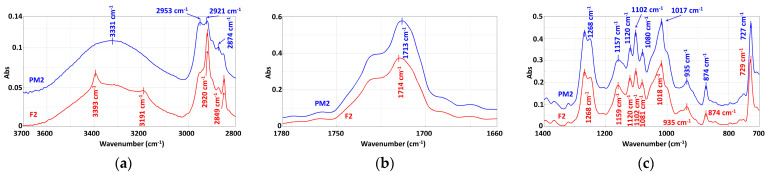
The FTIR spectra of the F2 and PM2 compounds: (**a**) 3700–2800 cm^−1^; (**b**) 1780–1680 cm^−1^; (**c**) 1400–700 cm^−1^.

The primary compound F4 had a glass transition at 55.6 °C that started at 46.8 °C and ended at 60.2 °C, but the mechanically recycled blendPM4 did not have such a transition (Figure 7, Table 4). While the primary compound F4 had a single melt with a maximum at 113.9 °C thatstarted at 86.2 °C and required 6.78 J/g (Figure 8a, Table 5), the mechanically recycled blend PM4 had two almost successive melting intervals, one at 74.5 °C that started from 30.2 °C with a caloric effect of 2.77 J/g, and a second one with a maximum at 122.7 °C that started almost immediately after the first melt at 78.8 °C and required 11.32 J/g (Figure 7, Table 6). Comparing the characteristics of the glass transition of the primary compounds F4 (Figure 7a, Table 4) and those of the mechanically recycled PM4 (Figure 7a, Table 6), which was produced in the negative temperature range, it can be assumed that they were just about similar. The process started at −41.6 °C and ended at approx. −30.9 °C, with T_g_ having a value of −36.2 °C and ΔC_p_being 0.19 g∙°C for F4, and 36.2 °C with a ΔC_p_ of 0.19 g∙°Cfor PM4.

The F4 compound crystallized at 61.5 °C in a process that started at 70.1 °C and released 9.62 J/g (Figure 7c, Table 4), while the PM4 compound crystallized at 103.4 °C, i.e., at a 42 °C higher temperature, starting at 115.8 °C and releasing8.39 J/g (Figure 7c, Table 7). The F4 compound lost 2.31% of its weight up to 175 °C with a maximum at 118.4 °C. Between 175 °C and 340 °C it lost 22.9% with a maximum at 305.3 °C and 67.43% between 340 °C and 465 °C with a maximum at 406.3 °C. Between 465 °C and 530 °C, this compound lost 0.67% more, and between 530 °C and 720 °C it lost0.67% of its weight. The residue at 720 °C was5.58% in nitrogen and 0.37% in air. By comparison, the mechanically recycled compound lost 1.89% up to 175 °C with a maximum at 118.4 °C, 22.9% between 175 °C and 340 °C with a maximum at 340 °C, and 43% between 340 °C and 465 °C with a maximum at 406.3 °C. Furthermore, when the temperature increased, an additional 1.11% was lost between 465 °C and 530 °C, 0.67% between 530 °C and 720 °C, and the residue at 720 °C was 2.1% in nitrogen and 1.98% in air.

All these results demonstrated that the PET-CW from which the recycled pellets were obtained degraded during storage in uncontrolled conditions, mainly through a mechanism of breaking macromolecular chains and the destruction of intermolecular attractions, which can fully explain the observed differences between the thermal behaviors of the mechanically recycled compounds. Because of the short chains resulting from the breaking of the macromolecules, which have a higher mobility, the mechanically recycled compounds melted and crystallized at higher temperatures. The destruction of intermolecular attractions through storage degradation generated a partial compositional de-mixing that was observed for all the studied mechanically recycled compounds. The de-mixing was partial because at negative temperatures, the primary compounds had approximately the same glass transition as the mechanically regenerated ones, at approximately −35 °C. This value was closer to the T_g_ of PBAT (−30 °C) [28], the majority component, and further away from that of PCL (−50 °C) [29], the minority component in all the studied compounds. The crystallization of the chains resulting from degradation causes the weight ofthe recycled compounds, even up to 175 °C, to be reduced. Other transformations were related to dehydrogenation and cyclization reactions, and pyrolysis, which would explain the TGA behavior with the formation of more stable compounds and the quantity of the residue in nitrogen and in air, for the mechanically recycled compounds in comparison with the primary ones.

The difference between the residues in the air at 750 °C of the mechanically recycled compounds PM2–PM5 and the residues in the air of the primary compounds F2–F5 represents the stable compounds resulting from the thermal destruction of the polymeric components of the mechanically recycled pellets degraded during storage. These residues were 5.25% for PM2, 1.78% for PM3, and 0.59% for PM4.

### 3.3. Chemical Structure

**Figure 9:**
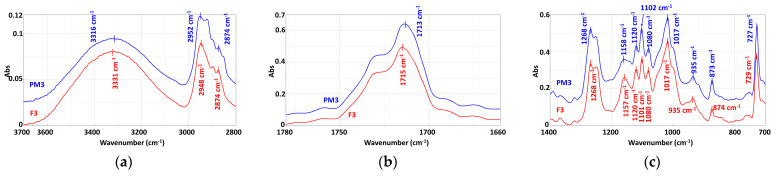
FTIR spectra of the F3 and PM3 compounds: (**a**) 3700–2800 cm^−1^; (**b**) 1780–1680 cm^−1^; (**c**) 1400–700 cm^−1.^

**Figure 10:**
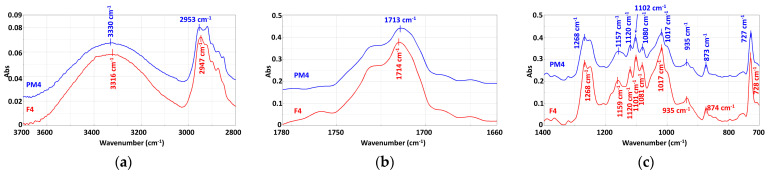
FTIR spectra of the PM4 and F4 compounds: (**a**) 3700–2800 cm^−1^; (**b**) 1780–1680 cm^−1^; (**c**) 1400–700 cm^−1.^

The comparative analysis of the FTIR spectra of the mechanically recycled compounds with the primary ones (Figure 8, Figure 9, Figure 10 and Table 11) is described below. Their normalized versions (Figures S1–S3) are presented in the Supplementary Materials section.

**Table 11:** Wavelengths of FTIR peaks of the analyzed compounds, their assignments, and those of the constituent polymers of these compounds [23,24,25].

Functional Group	Wavenumber, cm^−1^
a. From compounds	
−OH –NH and/or –NH_4_^+^	3394 cm^−1^, 3335 cm^−1^, 3313 cm^−1^, 1631 cm^−1^, 652 cm^−1^, 647 cm^−1^, 615 cm^−1^, 611 cm^−1^ 3200 cm^−1^
–CH_3_	2953 cm^−1^, 2874 cm^−1^
–CH din –CH_2_	2921 cm^−1^, 2850 cm^−1^
C=O	1754 cm^−1^, 1725 cm^−1^ shoulder, 1713 cm^−1^
–C=C– unsaturated	1646 cm^−1^, 1631 cm^−1^
–C=C– unsaturated from the aromatic ring –Aromatic ring	1578 cm^−^1505 cm^−1^
–CH from –CH_2_ –CH_2_ and/or –O–CH	1467 cm^−1^, 1454 cm^−1^, 1410 cm^−1^, 1392 cm^−1^, 1366 cm^−1^ 1321 cm^−1^
–C–C(O)–O	1269 cm^−1^, 1252 cm^−1^
–C–O–C and/or –(O)–OH –CH aromatic C–O	1252 cm^−1^, 1160 cm^−1^, 1119 cm^−1^ 1103 cm^−1^, 1080 cm^−1^, 868 cm^−1^
–C–CH_3_ and/or C–OH –C–C –C–O –C–C– –CH_2_ of chain	1080 cm^−1^, 1027 cm^−1^, 1018 cm^−1^, 935 cm^−1^, 759 cm^−1^ 1018 cm^−1^ 936 cm^−1^ 868 cm^−1^ 874 cm^−1^ 729 cm^−1^
**b. From the compounds’ components**	
Starch [23]	3600–3300 cm^−1^ OH; 2931 cm^−1^ C–O C–O 1637–1458 cm^−1^ CH_2_ 1385–1375 cm^−1^ C–H1149 cm^−1^ C–O–C 1200–800 cm^−1^ C–O 920 cm^−1^ 856 cm^−1^ 758 C cm^−1^–O–C ring vibration
PCL [24]	3600 cm^−1^–3400 cm^−1^ -OH; 3000 cm^−1^–2800 cm^−1^–CH3, CH_2_, CH; C=O 1730 cm^−1^
PBAT [24,25]	3000 cm^−1^ alcohol OH; 1735 cm^−1^–1725 cm^−1^ C=O from ester; 1256 cm^−1^ C-O from ester; 901 cm^−1^–700 cm^−1^ benzene ring, 726 cm^−1^ 4 adjacent –CH_2_–
Carbon black [26]	–COOH at 1720 cm^−1^, –C(O) –O–C(O), –(–C(=O)–O) 1735 cm^−1^, –C(O) –H 1700 cm^−1^
Phthalocyanine [25]	NH AT 3289 cm^−1^ 1006 cm^−1^, 3074 cm^−1^ 3048 cm^−1^–CH asymmetric, 2923 cm^−1^ 2854 cm^−1^–CH symmetric, 1609 cm^−1^ C–C in pyrrole, 1428 cm^−1^ 1332 cm^−1^ C–C in isoindole 625 macro cycle ring 1517 cm^−1^ 1490 cm^−1^ 1478 cm^−1^ C–H in aryl 1184 cm^−1^ 1164 cm^−1^ 1070 cm^−1^ C–N in isoindole

#### 3.3.1. Mechanically Recycled Compounds from the Pre-Consumer Waste from the Manufacture of Films Flexographically Printed with Black (PM2) and from the Primary Pellets Used in this Production (F2) (Figure 8)

In the spectral range of 3600 cm^−1^–3000 cm^−1^ of the mechanically recycled and primary compounds, two well-defined peaks appeared, one for –OH at 3400 cm^−1^ and then another forNH at 3203 cm^−1^ and 3193 cm^−1^. The –OH is related tothe high starch content of such functional groups, but also tothe-OH in the end-chains of PCL and PBAT, the other two polymers from the studied compounds. The –NH absorptions are due to the light-stabilizer type hindered amine that was used. These absorptions were not found in the spectrum of the recycled PM2 compound, possibly due to their involvement in a blocking mechanism of the hydroperoxides formed during the storage degradation of PET-CW, as explained in chapter 4. Otherwise, the FTIR spectrum of the PM2 compound in this range had a broad, uniform absorption, with a slightly larger surface area than F2, which could be a proof of the increase in –OH as a consequence of the storage degradation. In the 3000–2800 cm^−1^ range of the F2 spectrum, there is a slightly high, well-defined peak at 2920 cm^−1^ and another smaller one at 2849 cm^−1^, and the PM2 in this spectral range had a broad absorption of low intensity with several shoulders. The absorptions from 2920 cm^−1^ and 2849 cm^−1^ indicate vibration of the –CH from –CH_2_. The change occurring for the PM2 compound at these wave numbers can be proof that hydrogen was involved in interactions that did not exist in the primary compound (Figure 8a). In the 1780 cm^−1^–1680 cm^−1^ range, the absorption at1716 cm^−1^forthe C=O group from PCL and PBAT (Table 10) had two shoulders at 1754 cm^−1^ and 1712 cm^−1^. This peak was more intense for the mechanically recycled compound (Figure 8b). The absorption of low intensity from 1646 cm^−1^to 1505 cm^−1^ indicates the presence of an aromatic from PBAT and was more intense for F2, while it significantly decreased in intensity for the mechanically recycled PM2 compound (Table 11), possibly due to the degradation processes underwent by the PET-CW during storage. The absorptions generated by the vibrations of –CH from –CH_2_ at 1467 cm^−1^, 1410 cm^−1^ and 1366 cm^−1^ in the F2 spectrum were more intense than in those of the mechanically recycled compound. Another small change that appeared in the spectrum of the mechanically recycled PM2 compound regards the absorption at1158 cm^−1^corresponding to glycosidic linkage from starch (Table 11), whose peak wasslightly flatter than for the F2 primary compound (Figure 8c). All the changes presented above from the mechanically recycled spectra indicate the involvement of starch and the other two polymers, PCL and PBAT, in certain destruction reactions, other than chain breaks. All the absorptions from 1400 cm^−1^–700 cm^−1^ of the F2 compound were also found in the recycled compound spectrum, but as less intense versions (Figure 8c). The peak at726 cm^−1^ characterizing the four adjacent –CH_2_ from PBAT (Table 10) was more intense in the mechanically recycled compounds than in the primary ones.

#### 3.3.2. Mechanically Recycled Compounds from Pre-Consumer Waste Formed from Manufacture of Films (PM3) and Primary Pellets (F3) Used in this Production (Figure 9)

The comparative evaluation of the PM3 and F3 compounds’ spectra showed the same absorptions in both materials but with much sharper peaks for the mechanically recycled type, which indicates a higher mobility of the macromolecules in this blend. Another difference recorded for the mechanically recycled compound was the slight flattening of the broad absorption between3600 cm^−1^–3000 cm^−1^, which suggestsa smaller number of –OH groups involved in the absorption, possibly due to the slight degradation during storage of the starch from the composition of this blend (Figure 9a). Another change found in the PM3 spectrum wasthe increased intensity of the absorption peak at 1715 cm^−1^ of C=O, from PCL and PBAT (Table 11), which also means that this functional group has a greater mobility. The absorptions at1504 cm^−1^, 1457 cm^−1^, 1410 cm^−1^, and 1366 cm^−1^were attributed to the vibrations of –CH from the –CH_2_ found in all threepolymers from the studied blends PCL, PBAT, and starch were more intense in the mechanically recycled compounds, possibly also due to the degradation of the PET-CW during storage (Figure 9b). The absorption peaks of the –C–C(O) –O, –C–O-C and/or –C(O) –OH functional groups at 1268 cm^−1^ and 1248 cm^−1^ from starch, PCL and PBAT (Table 10) also became slightly more intense for the mechanically recycled compound, possibly due to the storage degradation of the PET-CW, which generated a greater mobility of the macromolecular chains, the short chains being more mobile than the longer ones. Another change found in the PM3 spectrum wasat1158 cm^−1^ where the glycosidic linkage from starch had a slightly flatter peak (Figure 9c), indicating the added effect of starch during storage degradation, which was also proven by the changes in the area of the –OH absorptions. The 726 cm^−1^ peak assigned to the four adjacent –CH_2_– from PBAT (Table 10) was more intense in the mechanically recycled compound PM3, probably also because of the PET-CW storage degradation.

#### 3.3.3. Mechanically Recycled Compound from Pre-Consumer Waste Formed from Mass Manufacture of Colored Film (PM4) and Primary Compound (F4) Used in this Production (Figure 10)

The FTIR spectrum of compound PM4 showed absorptions at the same wavelengths as in the primary compound F3, with the difference that the intensities of all peaks were lower (Figure 10). In the spectrum of the mechanically recycled compound, the following changes also appeared: the broadening of the –OH absorption from starch between 3600 cm^−1^ and 3000 cm^−1^; the flattening of the peak with shoulders from 2953 cm^−1^ and 2920 cm^−1^ assigned to the CH and CH_2_ vibrations for PCL, PBAT, and starch (Figure 10a); the decrease in the CO peak intensity from 1713 cm^−1^forPCL and PBAT (Figure 10b); a slight broadening of the peak from 1455 cm^−1^; the formation of a single peak with shoulders from 1456 cm^−1^ and 1366 cm^−1^ (Figure 10c) attributed to the– CH and –CH_2_of PCL, PBAT, and starch (Table 11). Another change in the mechanically recycled compound spectrum was the decrease in intensity and slight broadening of the peak at 1268 cm^−1^ assigned to the vibrations of –C–C(O)-O, –C–O-– and/or –C(O)–OH groups from PCL, PBAT, and starch (Table 11). The flattening of the 1159 cm^−1^ peak attributed to the vibration of the glycosidic bond from starch as well as the changes described for the -–OH group absorptions between 3600 cm^−1^ and 3000 cm^−1^ showed the starch degradation during PET-CW storage. The peak at 726 cm^−1^ assigned to the four adjacent –CH_2_– from PBAT (Table 10) was smaller in the mechanically recycled compound (Figure 10). The phthalocyanine dye absorptions (Table 11) were not identified in either the primary or the secondary compounds, probably because of too small quantities being used.

According to the above-presented FTIR, the spectra of the mechanically recycled compounds showed changes, but not in terms of the appearance of new peaks or the disappearance of others and/or peak translations, which if they existed would prove deep structural changes of a chemical nature [30].The changes that occurred in these spectra were mainly related either to an intensification of the absorption of all the characteristic peaks, as in the PM2 and PM3 compounds, or a decrease in these absorptions, as inthe PM3 blend. This type of transformation is related to the mobility of the macromolecular chains, which is associated with the length of the macromolecular chains. Another change regards the widening and flattening of some absorption peaks, described above, indicating the involvement of the particular functional groups of constituent polymers in chemical reactions other than chain cleavage. In all the mechanically recycled compounds’ spectra, there were changes in the area characterizing the starch absorptions at 3600 cm^−1^–3000 cm^−1^ for the –OH absorption and at 1256 cm^−1^ for the glycosidic linkage. Changes inthe functional groups from PCL and PBAT, the other two polymers from the studied compounds, were: C=O from PBAT and PCL at 1730 cm^−1^; benzene ring at 901 cm^−1^–700 cm^−1^; the four adjacent –CH_2_– from PBAT at 726 cm^−1^; the CH from CH_2_ from all the tree polymers at 3000 cm^−1^–2800 cm^−1^. No spectral changes were identified with respect to the carbon black with which the flexographic printing was performedin the case of the PM2 compound, or the phthalocyanine used for bulk coloring in the case of the F4 and PM4 blends.

The increase in the peak intensities found in the recycled PM2 and PM4 compounds could be explained by a greater kinetic independence of the macromolecular chains, made possible through the destruction of intermolecular attractions and the diminishing of the macromolecular chain lengths (short chains have greater mobility) during PET-CW storage. This type of structural change is usually followed by compositional de-mixing, which was otherwise illustrated by the melting behavior of all mechanically recycled compounds. The decrease in the intensity of all the absorption peaks for the recycled PM4compound, as well as the flattening of the absorptions of the functional groups of the three polymers from this compound, proved that in addition to chains breaking, the destructive mechanism during PET-CW storage also involved the functional groups from the three polymers, to a greater extent compared to the previous cases and therefore their number was reduced. It could be stated that the extent to which the functional groups specific to each polymer from the mechanically recycled compounds disappeared from the system was correlated with the destruction intensity during PET-CW storage.

### 3.4. Mechanical Properties

**Figure 11:**
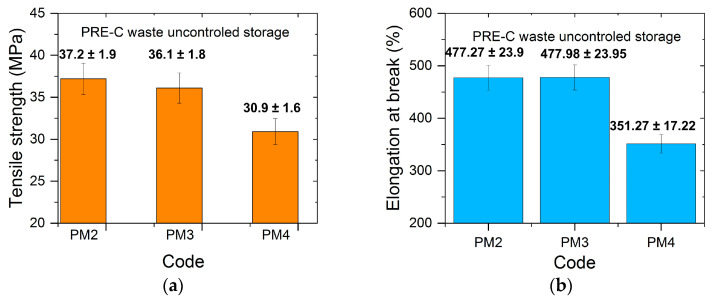
The mechanical properties of films with mechanically recycled compound content (PM2, PM3) from PET-CW stored in uncontrolled conditions ((**a**) Tensile strength at break; (**b**) elongation at break).

The mechanical properties of the newly obtained multilayer films with mechanically recycled compound content (PM2, PM4) showed scattered values regarding the tensile strength upon breaking, varying between 30.9 MPa and 37.2 MPa, and elongation upon breaking, from 351.27% to 477.98% (Figure 11). From the comparative analysis of the tensile strength properties of the films made from primary compounds (Table 2), which were compounded with mechanically recycled ones, it is clear that the lower values were registered in the first situation. However, a greater variation was observed for the break elongation, which is explainable if the degradation during storage mainly occurred through the macromolecules breaking, as described by the above-presented results.

From the practice of polymer chemical degradation, it is known that the reduction of mechanical properties is not synchronous with the moment when the first structural changes appear. For conventional polymers such as polyolefins, for example, correlations between structural changes and the diminishing of functional properties under the application requirements are known [31,32]. Generally, the functional properties fall below the application requirements after the accumulation of structural changes up to a certain level, which are reached following the end of the induction period [31]. If this correlation must be identified in each case for conventional polymers, then it is even more important for the compounds based on polymers of renewable origin. For the studied mechanically recycled compounds, the deviation of the mechanical properties from the primary compound levels was not significant. It might have been possible that if the PET-CW had been kept longer in the uncontrolled described conditions, then the structural changes caused by degradation during storage wouldhave been more pronounced, and therefore the influence on the mechanical properties would have been greater.

## 4. Discussion

All the obtained results lead to the idea that PRE-CW degrades during storage in uncontrolled conditions, with destruction processes possibly accelerated during mechanical recycling. The degradation is demonstrated by:

- the FTIR changes, which may have been a consequence of degradation via the breaking of macromolecular chains and of intermolecular bonds with more or less involvement of functional groups corresponding to each polymer from the studied compounds;
- the 2–5-times greater melt fluidity of all the mechanically recycled compounds, most likely being due to the PRE-CW degradation through chain breaking during storage, with the molecular weight decreasing;
- the melting of mechanically recycled polymers in successive processes, over the entire range of positive temperatures up to 120 °C, compared to a single melt, which had a relatively narrow end with a maximum at almost 120 °C. This behavior could be a result of the compositional de-mixing following the chain breaking and the alteration of the intermolecular interactions because of the storage degradation of the PET-CW;
- the crystallization of mechanically recycled compounds at 20 °C–35 °C higher temperatures than the primary compounds, possibly because of a large number of short chains appearing via chain breaking, which had a greater mobility and crystallized much more easily.
- the thermal degradation of the recycled compounds with the formation of slightly more stable compounds with the movement of the TGA degradation steps towards higher values and with a greater amount of residue at the end of the process, at 750 °C (stable compounds in air).

Starch, one of the polymers from the studied compounds, is a polysaccharide of renewable origin and of composite type because of the two immiscible components: amylose and amylopectin [33]. A main factor in the degradation of starch and of starch-based bioplastics is the hydrophilicity of the two component polymers, such that starch is very susceptible to hydrolysis at the glycosidic bond [34]. Under enzymatic conditions, this process generates biodegradation [35], which occurs via the destruction of the macromolecular chains to oligomers or monomers [34]. The temperature [36] intensifies the enzymatic hydrolysis [36]. Another characteristic destruction factor of the melt compounding techniques is the shear rate, which, in the case of starch, preferentially breaks the branches of amylopectin [37] rather than the amylose chains, as well as the crystalline areas of amylopectin, which are more sensitive to shear than the amorphous, flexible amylose structures [33,38]. Thermal degradation starts at 280 °C with condensation reactions between the hydroxyl groups of the neighboring chains and with the formation of ether bonds. Under extreme temperatures, the starch dextrinization takes place with the formation of levoglucosan and various volatile products [39]. These reactions that take place in starch under the influence of temperature can be an explanation for the behavior at high temperatures of the mechanically recycled compounds and of the residues formed.

PCL is another component of the studied compound, which is a biodegradable polyester of conventional origin and susceptible to hydrolysis [40]. The PCL degradation follows abiotic (chemical) or biotic (enzymatic) hydrolysis by cleavage of the ester bond, the C=O group being hydrolyzed and the -C-O- linkage being broken, a mechanism which generates a decrease in the molecular weight of the assembly and the appearance of functional groups such as hydroxide or carboxyl end-chains [41,42,43]. The thermal degradation of PCL occurs through a complex radical mechanism of random breaking of the macromolecular chains. This mechanism is controlled by the decomposition of hydroxyl or acid groups from the end of the macromolecular chains. The nature of the end-chain groups of PCL determines not only the stability during processing but also the stability during storage. It should also be mentioned that PCL is a polymer that melts at around 60 °C [44].

The biodegradable aliphatic aromatic co-polyester PBAT is another component of the studied compounds. Because of its ester bond content, it is hydrolysable through chemical or enzymatic mechanisms boosted by bacteria, fungi, and algae existing in the environment, as well as temperature and shear rate [45].

The degradation during the processing of PBAT occurs via hydrolysis of the ester bond through a predominant mechanism of chain breakage and β-C-H hydrogen transfer. The process cannot be stopped by using a stabilizer, with drying being a safe way to control the destruction [46]. The presence of starch in the studied compounds increases the biodegradation rate by intensifying the hydrolysis reactions for both PCL and PBAT [47,48,49,50,51]. Consequently, all three polymers of the studied compounds are hydrophilic polymers susceptible to chemical hydrolysis. If the hydrolysis takes place under enzymatic conditions, then well-known structural damage occurs with a loss of integrity and transformation into CO_2_ and water, i.e., biodegradation [52,53,54,55].

It follows that the studied compounds, which contain biodegradable polymers due to the content of glycosidic bonds (in the case of starch) and ester bonds (of PCL and PBAT), have a very high susceptibility to hydrolytic degradation. Considering the storage during hot, rainy summers with many alternating heat and rain periods, in closed, unventilated spaces where microorganisms develop and the temperatures reach 60 °C–70 °C, it is very easy to understand that, in this way, conditions are created for enzymatic hydrolysis boosted by temperature, which is similar to the well-known composting conditions. Other factors that potentiate the bio-destruction of the studied compounds are the presence of starch, which can lead tothe destruction of the other two polymers from the compounds. Additionally, the low melting temperature of PCL of approx. 60 °C should not be forgotten. It is quite likely that the destruction begins on the PET-CW surface, at the points where microorganisms find PCL and starch, allowing it spread into the PET-CW mass. Due to the degradation by hydrolytic attack, potentiated by the microorganisms and temperature, of the glycosidic bond from the starch and/or of the esters from the two biodegradable polyesters, the macromolecular chains are broken. Depending on the intensity of the destructive attack, the changes that appear can mean only the breaking of the attraction between the macromolecules and of the macromolecular chains with the involvement of a small number of glycosidic or ester groups. The main consequence of these transformations is the partial compositional de-mixing of the compounds, as was observed in all the studied mechanically recycled compounds. The destructive hydrolytic attack can also mean the massive involvement of glycosidic and esters linkages from the three polymers of each mechanically recycled compound, which generates a severe destruction with the loss of polymer characteristics. The size of the changes that appear in the FTIR spectra depends on the intensity of the described processes. It is understandable that because of such destructive processes, the compounds’ properties are affected both in the molten and solid states, the degraded compounds being much more fluid and having different thermal behavior, breaking properties, etc. It has been proved not only that humidity and temperature are extremely dangerous for these compounds, but also that the longer the storage time in these conditions, the more pronounced the destruction process [40,56,57].

If these wastes are stored for a long time, especially in uncontrolled conditions, then the chemical destruction can be potentiated by macromolecular relaxation, especially when the compound was frozen in a thermodynamically unstable state. The macromolecular relaxation determines the well-known physical aging of polymeric materials (which should not be associated with chemical aging), or storage aging [58]. If the material was frozen in an energetically unstable state, then, due to the relaxation, the macromolecules continuously change their conformation until reaching the ball state, when the functional properties of the material as a whole no longer resemble those required. The faster the thermodynamic balance changes, the sooner the functional properties are lost. To comply with the application requirements, the storage time until use must not be longer than the thermodynamic equilibrium of the polymeric materials [31,32,59,60].

It is obvious that in order to avoid the chemical and physical degradation of PRE-CW, they must be immediately mechanically recycled without storage. Otherwise, they must be kept in spaces with controlled temperatures of 4 °C–20 °C, preferably at 4 °C, andin the case of film fabrication, not for more than three months. Such an approach means the development of sustainable (covers certain consumer values without endangering the environment, health and the economy or affecting the abilities of the future generations to ensure their requirements), re-thought (PRE-CW mechanically recycled into good-quality films) packaging fabrication that is well-integrated into the circular economy.

The coloring does not induce spectacular differences between the fluidity of the mechanically recycled compounds compared to the primary ones. For bulk dyeing, the fluidity of the mechanically recycled compounds is similar to non-dyed mechanicallyrecycled compounds. Flexographic printing with carbon black does not induce other variations compared to the recorded uncolored mechanically recycled compounds.

In the competition for the efficiency of the mechanical recycling of compounds based on starch and biodegradable polyesters for films, the elimination of PET-CW storage and their immediate mechanical recycling has a decisive role. Other important factors in increasing the efficiency of mechanical recycling of hygroscopic polymeric materials are drying before melt-processing, as they are hygroscopic compounds, the selection of working conditions so as to harmonize the needs of the transformation into the finished product in protected conditions (low and medium temperatures, medium shear speeds) with economic consequences.

## 5. Conclusions

1. It was demonstrated that the pre-consumer waste (PRE-CW) from the manufacture of multilayer packaging films starting from starch-, PCL-, and PBAT-based compounds cannot be stored before mechanical recycling, especially in uncontrolled conditions.In spaces without ventilation, during the hot, rainy summers with alternating scorching heat and rain in these unventilated spaces, conditions similar to composting are created. Temperatures around 50 °C–60 °C enable enzymatic hydrolysis on the PET-CW surface, probably at the glycosidic linkages from starch and the esters from PCL (a polymer with a melting temperature of 60 °C) and PBAT. Once the sitesof destruction are created, then it propagates into the mass. Storage time accelerates the destruction that is also possibly due to the physical aging, which in turn can be enhanced by chemical degradation.
2. The degradation was highlighted by:
  - the FTIR changes, which may be the consequence of some destruction processes, with the breaking of the macromolecular chains’, destruction of intermolecular bonds and/or more or less involvement of functional groups corresponding to each polymer from the studied compounds;
  - the 2–5-times greater melt fluidity of all the mechanically recycled compounds, which was most likely due to the PET-CW degradation during storage with the molecular weight decreasing through chain breaking;
  - the melting of mechanically recycled compounds in successive processes over the entire range of positive temperatures up to 120 °C, compared to melting with a single, relatively narrow endotherm with a maximum at almost 120 °C. This behavior can be a result of the partial compositional de-mixing following the alteration of the intermolecular interactions and/or the chain breaking, and of the storage degradation of PET-CW;
  - the crystallization of mechanically recycled compounds at 20 °C–35 °C higher temperatures than the primary compounds, which was possible because of the large number of short chains appearing via chain breaking, which had a greater mobility and crystallized much more easily;
  - the thermal degradation of recycled compounds with the formation of slightly more stable compounds with the movement of the TGA temperature steps towards higher values and with greater amounts of residues at the end of the process, at 750 °C (stable compounds in air).
3. The coloring did not introduce spectacular differences between the studied properties of the mechanically recycled pellets compared to the primary ones, possibly because of the very small amounts of dyes used. The flexographic printing with carbon black did not induce variations other than those recorded in the case for the uncolored recycled compound.
4. In order to make the mechanical recycling of starch- and biodegradable-polyester-based compounds for packaging films more efficient, the PET-CW must be immediately mechanically recycled without storage. This method avoids both chemical degradation and the physical aging, which can reciprocally potentiate each other much more the longer the storage time.
5. Mechanical recycling can also be made more efficient by drying the primary granules prior to mechanical recycling, and/or through the optimal selection of working conditions to ensure protection (low and medium temperatures, medium shear speeds) and the immediate recycling of waste.

## Figures and Tables

**Table 1:** Mechanically recycled pellets from PRE-CW.

Code	Fraction of PRE-CW after Color Separation	Coloring	MFI ^x^, g/10 min
PM2	Flexographic printing with black	Carbon black	10.1
PM3	Unprinted, uncolored	-	14.2
PM4	Bulk coloring	Phthalocyanine blue	14.8

^x^ 160 °C/5 kg.

**Table 2:** Compounds used in primary film manufacturing (STARCH, PBAT, PCL compounds) ^x^.

Code\Properties	Properties of Primary Compounds ^2x^				
	Density (ρ) g/cm^3^	Fluidity (MFI) ^3x^, g/10 min	Melting Temp., °C	Break Tensile Strength, (*σ_br_*), MPa	Elongation at Break, (ε), %
F2	1.29	3	118	30	450
F3	1.27	4.5	117	35	485
F4	1.28	3	115	30	390

^x^ undisclosed composition; ^2x^ measurements according to methods presented in the Section 2.3.3; ^3x^ 160 °C/5 kg.

**Table 3:** The variation of the fluidity increment in the ranges of 130 °C–170 °C and 2.16–10 kg load for mechanically recycled pre-consumer wastes and primary compounds.

CODE	The Variation of the Fluidity Increment in the Ranges of 130 °C–170 °C and 2.16–10 kg Load for Mechanically Recycled Pre-Consumer Wastes and Primary Compounds			
	g/10 min		g/10 min	
	2.16 kg–5 kg	10 kg	2.16 kg–5 kg	10 kg
I. Films with surfaceflexographically printed with black pigments				
PM2	1–8	13–13.5	140–150 °C: 2–5 160–170 °C: 2–6	140–150 °C: 9–10 160–170 °C: 2–14
F2	1–4	140–150 °C: 12 160–170 °C: 1–4	140–150 °C: 1.5–4.5 160–170 °C: 1–2	140–150 °C: 1–2 160–170 °C: 10–11
II. Colorless film				
PM3	1–6	13.5–16	0.5–6	4–16
F3	0.5–2	7.5–9.5	0.2–2	1.5–4.5
III. Mass colored films (green pigments)				
PM4	1–6	11.5–18	1–6.5	5.5–18
F4	0.5–5.5	11–15	0.9–6	5–8.5

## Data Availability

The data presented in this study are available on request from the corresponding author.

## Supplementary Materials

The following supporting information can be downloaded at: https://www.mdpi.com/article/10.3390/ma16041503/s1, Figure S1: The normalized FTIR spectra of the F2 and PM2 compounds; Figure S2: The normalized FTIR spectra of the F3 and PM3 compounds; Figure S3: The normalized FTIR spectra of the F4 and PM4 compounds.
