# Supplementary material for: New Biodegradable Materials for Re-Thought Packaging from Pre-Consumer Wastes by Controlling the Storage Time as Method to Increase the Mechanical Recycling Efficiency
Source: Materials (Basel). 2023 Feb 10;16(4):1503. doi: 10.3390/ma16041503 (PMC9960796; doi:10.3390/ma16041503)
Supplement: Supplementary file 1 [file materials-16-01503-s001.zip › materials-2179333-supplementary.pdf]

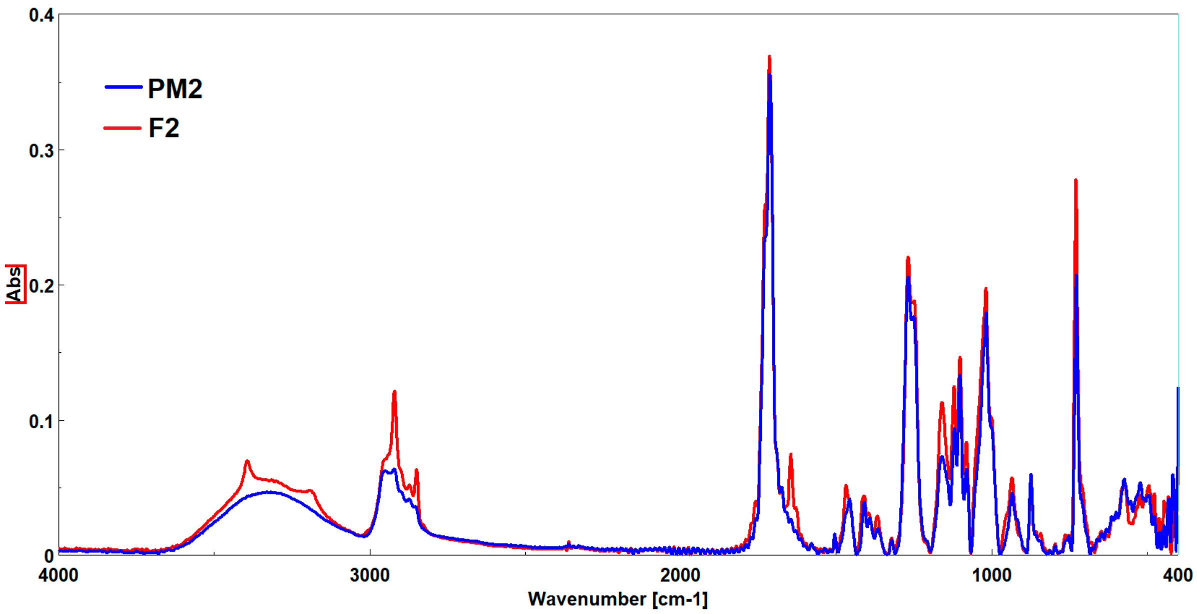

Figure S1. The normalized FTIR spectra of the F2 and PM2 compounds

each

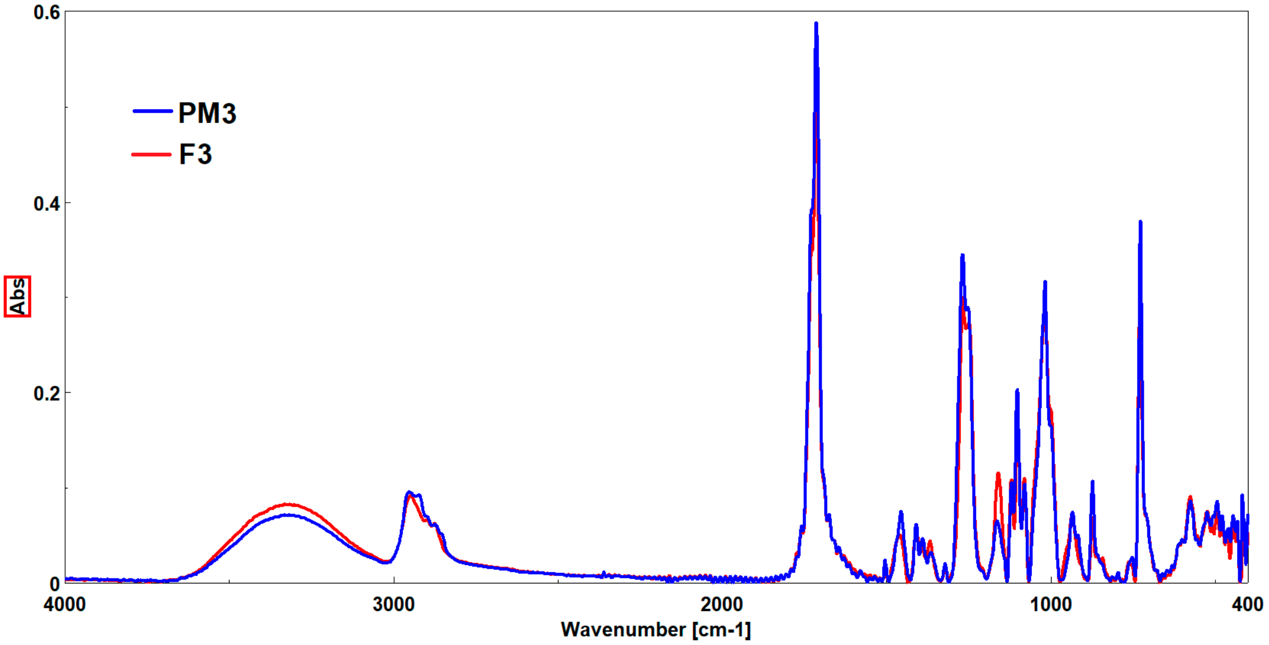

Figure S2. The normalized FTIR spectra of the F3 and PM3 compounds

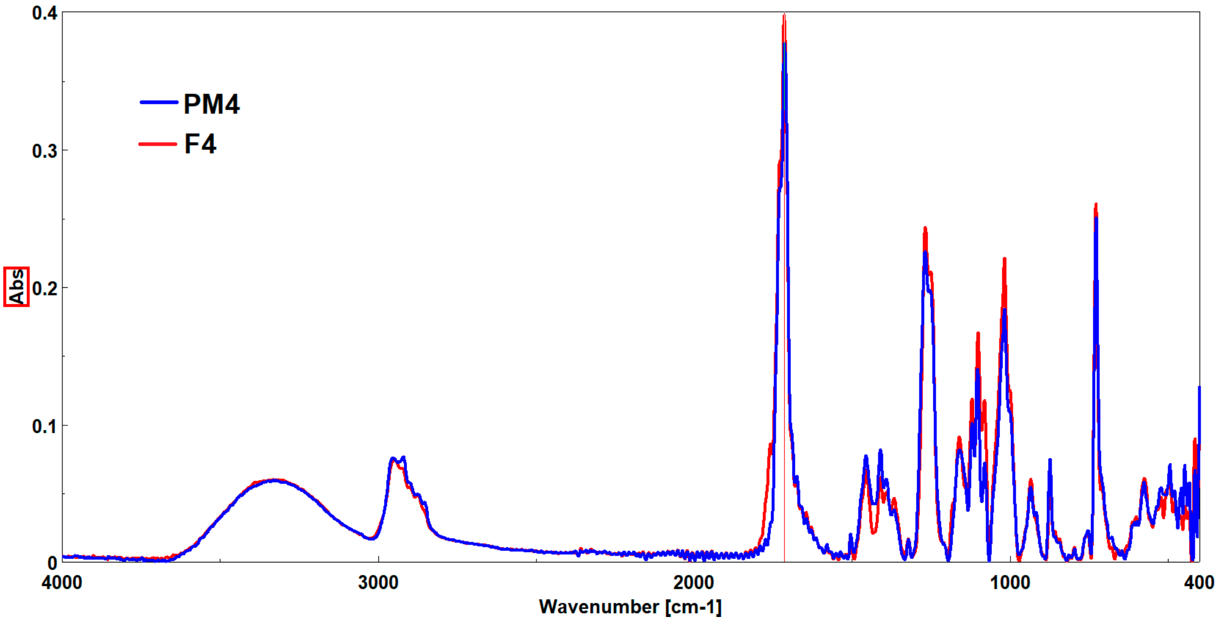

Figure S3. The normalized FTIR spectra of the F4 and PM4 compounds
